# Supplementary material for: ATM phosphorylation of the actin-binding protein drebrin controls oxidation stress-resistance in mammalian neurons and C. elegans
Source: Nat Commun. 2019 Jan 30;10:486. doi: 10.1038/s41467-019-08420-w (PMC6353951; doi:10.1038/s41467-019-08420-w)
Supplement: Supplementary file 2 — Supplementary Information [file 41467_2019_8420_MOESM2_ESM.pdf]

ATM phosphorylation of the actin-binding protein drebrin controls oxidation stress-resistance in mammalian neurons and *C. elegans*

Kreis et al.

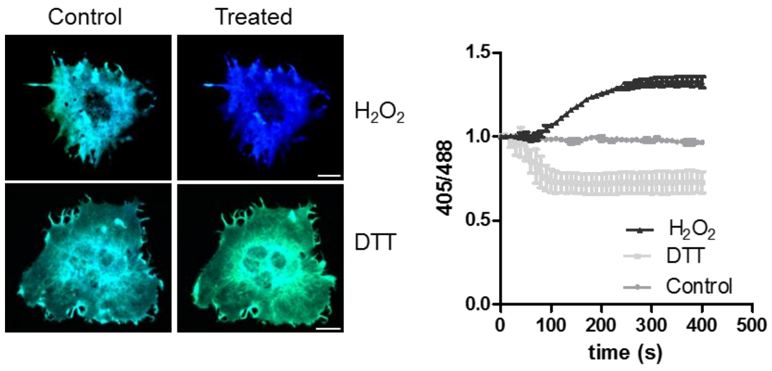

Supplementary Figure 1. Validation of the DBN-roGFP construct in monitoring redox changes

COS cells were transfected with DBN-roGFP and imaged 24 h later using spinning disk microscopy. Cells were treated with 0.1 mM H_2O_2, 7 mM DTT or medium alone (control).

Left: representative images resulting from excitation at 405 nm (false-coloured in blue) and from excitation at 488 nm (false-coloured in green) and represented as overlays. Scale bar 20µm. Right: mean ratio traces +/- SEM of emission intensities of 3 independent cells excited at 405 nm or 488 nm.

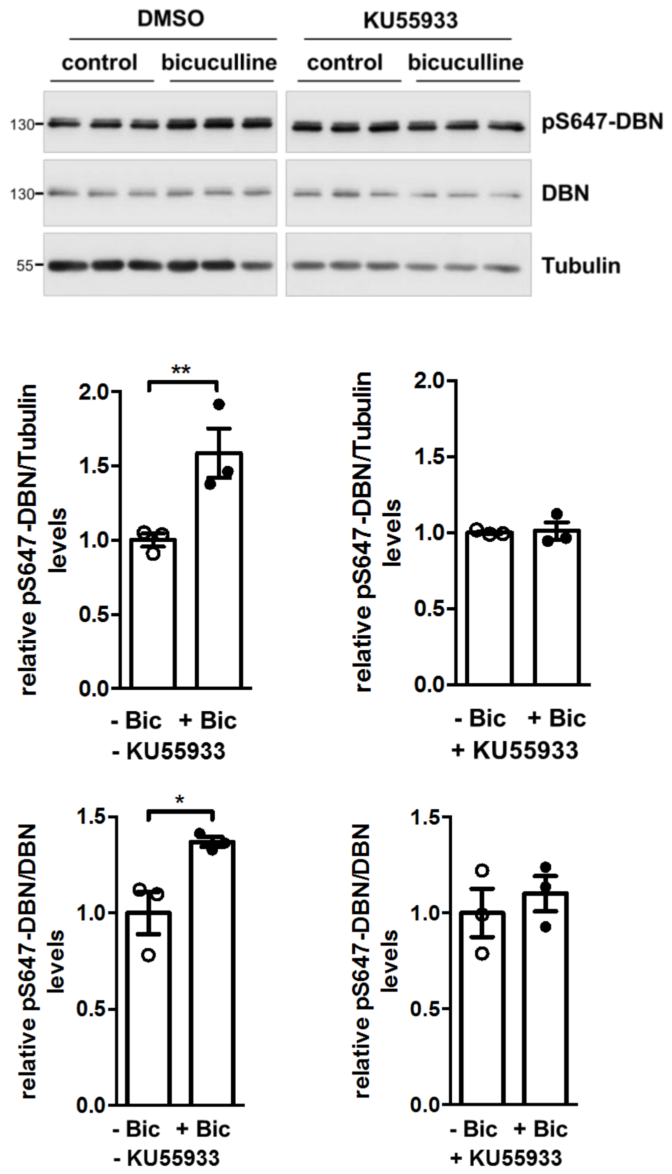

Supplementary Figure 2. ATM inhibition abolished bicuculline induced DBN phosphorylation at S647

Hippocampal rat neurons were treated with bicuculline (30 µM) for 1 h in the presence of DMSO (-) or 10 µM ATM inhibitor (KU55933) (+). Bar graph shows the relative band density of pS647-DBN/Tubulin and pS647-DBN/DBN in 3 biologically independent samples +/- SEM; One way ANOVA Bonferroni test. **p<0.01

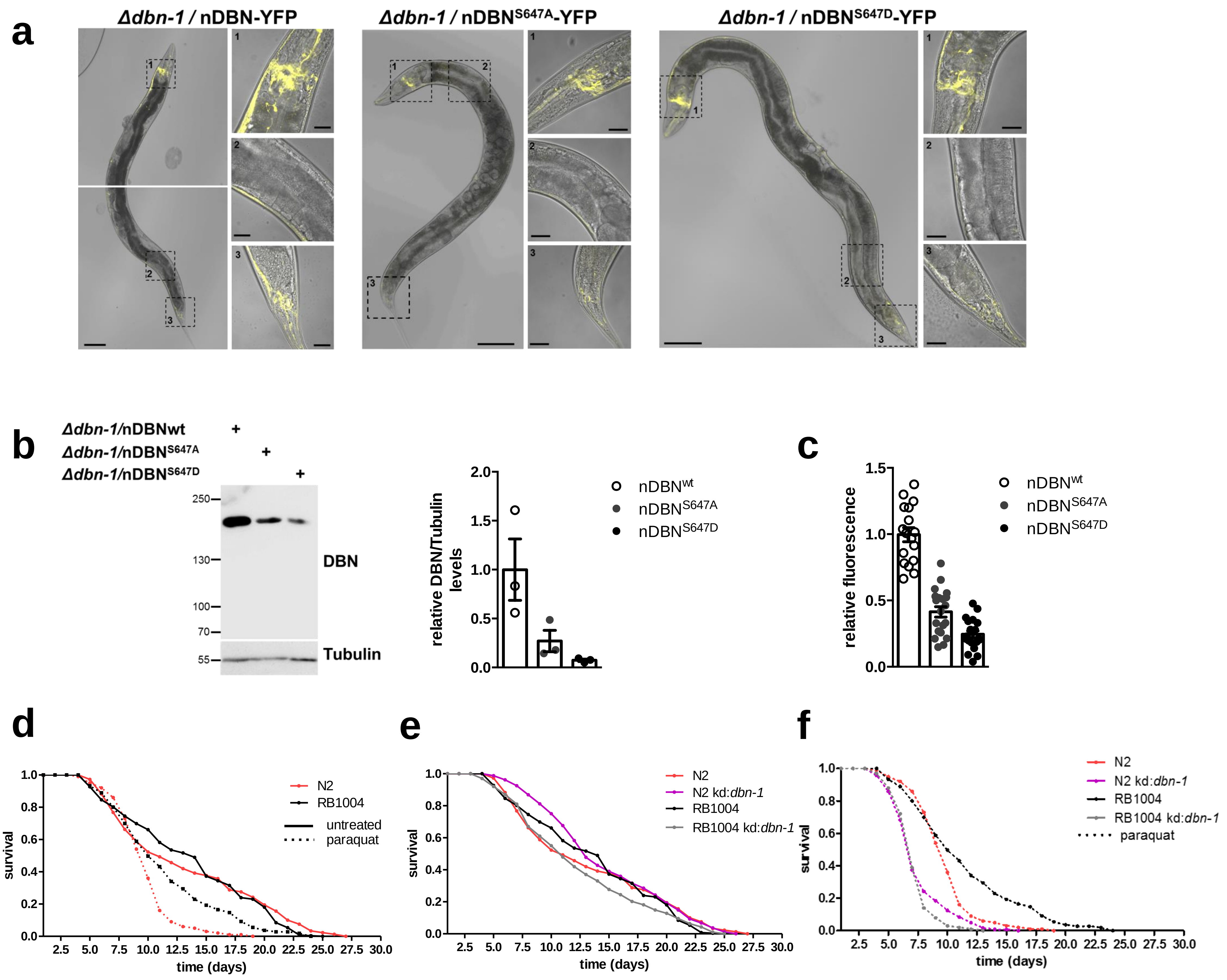

Supplementary Figure 3. Characterisation of Δdbn-1 C. elegans lines

Characterisation of wild type (N2), partial knockout (RB1004) and complete knockout (JKM1) of dbn-1 upon knockdown of dbn-1 and treated with or without Paraquat to induce oxidative stress. N=number of cohorts, n=number of worms per cohort.

(a) Pan-neuronal expression of human DBN-YFP (nDBN), DBNS647A-YFP (nDBNS647A) and DBNS647D-YFP (nDBNS647D). Left: confocal images of entire nematodes with 3 insets magnified to show head (1), nerve cord (2) and tail (3). Scale bar 100 µm, inset scale bar 25 µm.

(b) Western blot of total lysates from strains nDBN, nDBNS647A and nDBNS647D detecting DBN-YFP fusion proteins at approx. 180 kDa using M2F6 Drebrin-antibody) and alpha-tubulin. Bar graph shows the relative band density DBN/Tubulin in 3 independent worm cohortsexperiments. Depicted is mean ± SEM.

(c) Quantification of relative DBN-variants expression in nDBN-YFP, nDBNS647A-YFP and nDBNS647D-YFP by measurement of the YFP-fluorescence in the head region of 17 - 20 individual nematodes per strain. Depicted is mean ± SEM. nDBNwt: N=1, n=17; nDBNS647A: N=1, n=20; nDBNS647D: n=1, n=20.

(d) Lifespan assay showing the cumulative survival probability of N2 and RB1004 treated with paraquat (dashed line, N2: N=1, n=101; RB1004: N=1, n=120) or untreated (continuous line, N2: N=1, n=150; RB1004: N=1, n=179). Cohort sizes = 100 – 150 nematodes.

(e) Cumulative survival probability of untreated N2 (red, N=1, n=150) and RB1004 (black, N=1, n=179) nematodes and dbn-1 RNAi-treated N2 (magenta, N=1, n=160) and RB1004 (grey, N=1, n=100).

(f) Cumulative survival probability of untreated N2 (red, N=1, n=101) and RB1004 (black, n=1, n=120) nematodes and dbn-1 RNAi-treated N2 (magenta, n=1, n=120) and RB1004 (grey, n=1, n=120), all treated with paraquat.

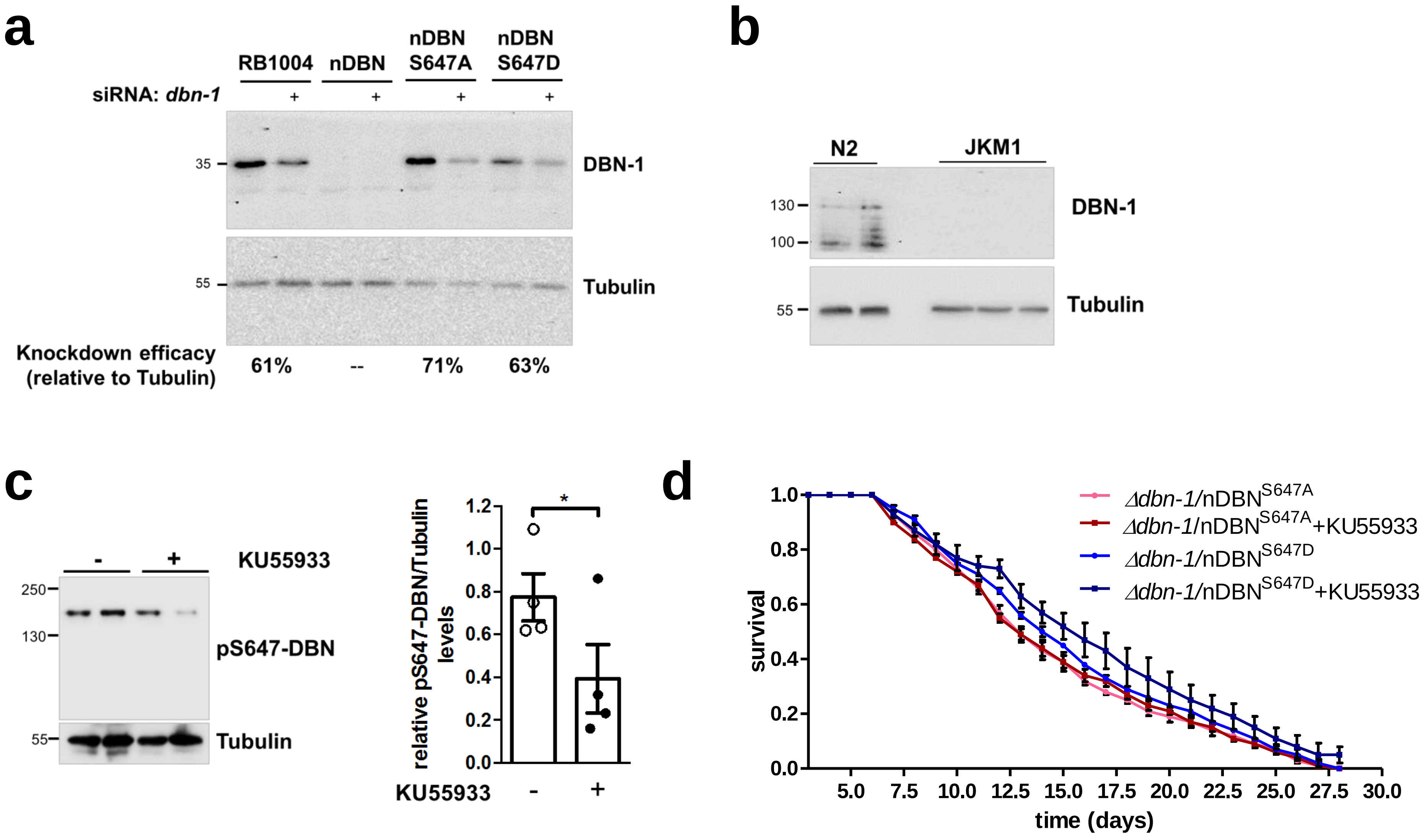

Supplementary Figure 4. Characterization of D*dbn-1 C. elegans* and efficacy of KU55933

(a) Verification of *dbn-1* knockdown in nDBN strains and in partial knockout strain RB1004 using no si-RNA or *dbn-1* si-RNA (+). Nematode lysates were analysed by western blot using a *C. elegans*-specific DBN-1 antibody to detect truncated nematode DBN-1 (35 kDa) and α-Tubulin serving as loading control. Two technical replicates were analysed for each condition. Knockdown efficacy was determined by measuring band density of DBN-1 over tubulin.

(b) Validation of the CRISPR/Cas9 generated knockout of *C. elegans dbn-1* by western blot. N2 lysates (two technical replicates) and JKM1 lysates (three technical replicates) were analysed using a *C. elegans*-specific DBN-1 antibody. As previously demonstrated[30], several bands were detected with DBN-1 antibody between 100 and 130 kDa in wild type animals (N2) (left). These bands were absent in the *dbn-1* knockout line (JKM1) (right).

(c) Reduction of pS647-DBN upon treatment with ATM kinase inhibitor KU55933. *C. elegans* Δ*dbn-1*/nDBN were synchronised, grown until L4 stage and treated with 1 mM KU55933 (+) or 2% DMSO (-) for 24 h. Left: Western blot analysis of nematodes lysates analysed by western blot using pS647-DBN and α-Tubulin antibodies. Right: Bar graph shows the relative band density of pS647-DBN/Tubulin of 2 technical replicates in 2 biologically independent experiments +/- SEM; T-test. *p<0.05.

(d) Lifespan analysis of phospho/dephospho-variant nDBN strains. Shown is the cumulative survival probability of nDBN^{S647A} and nDBN^{S647D} treated with or without ATM kinase inhibitor KU55933. Δdbn-1/DBNS647A: N=3, n=100-105; Δdbn-1/DBNS647D: N=3, n=90-106 where N represents the number of cohorts and n the number of worms per cohort.

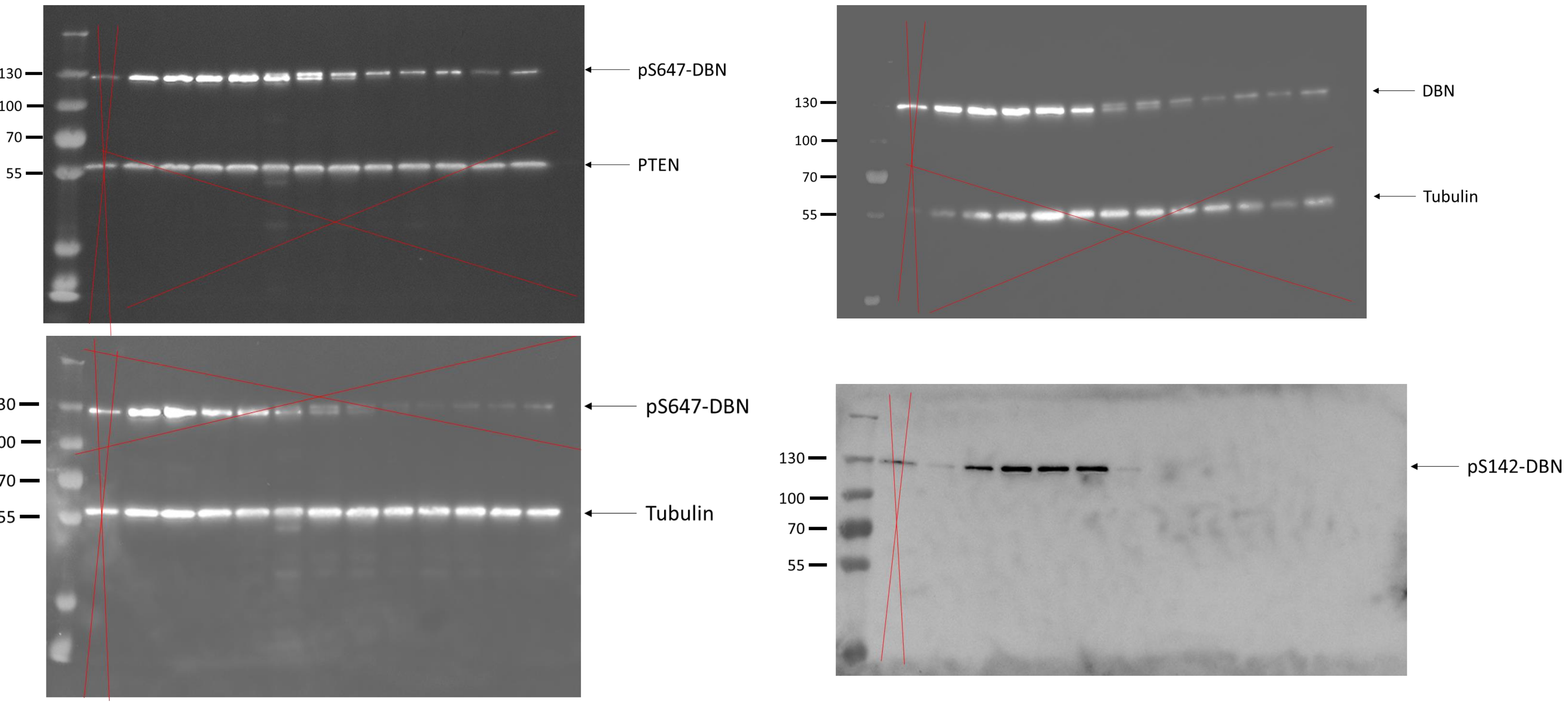

Supplementary figure 5. Uncropped western blots related to Figure 2a

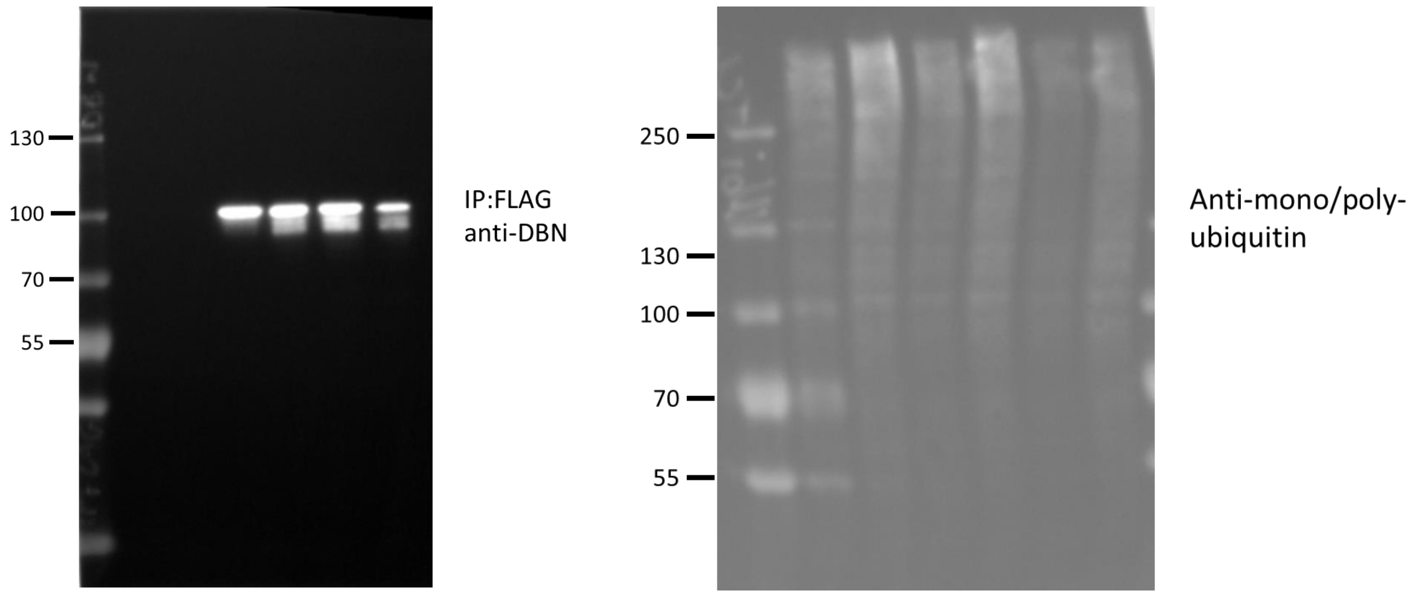

Supplementary figure 6. Uncropped western blots related to Figure 2f

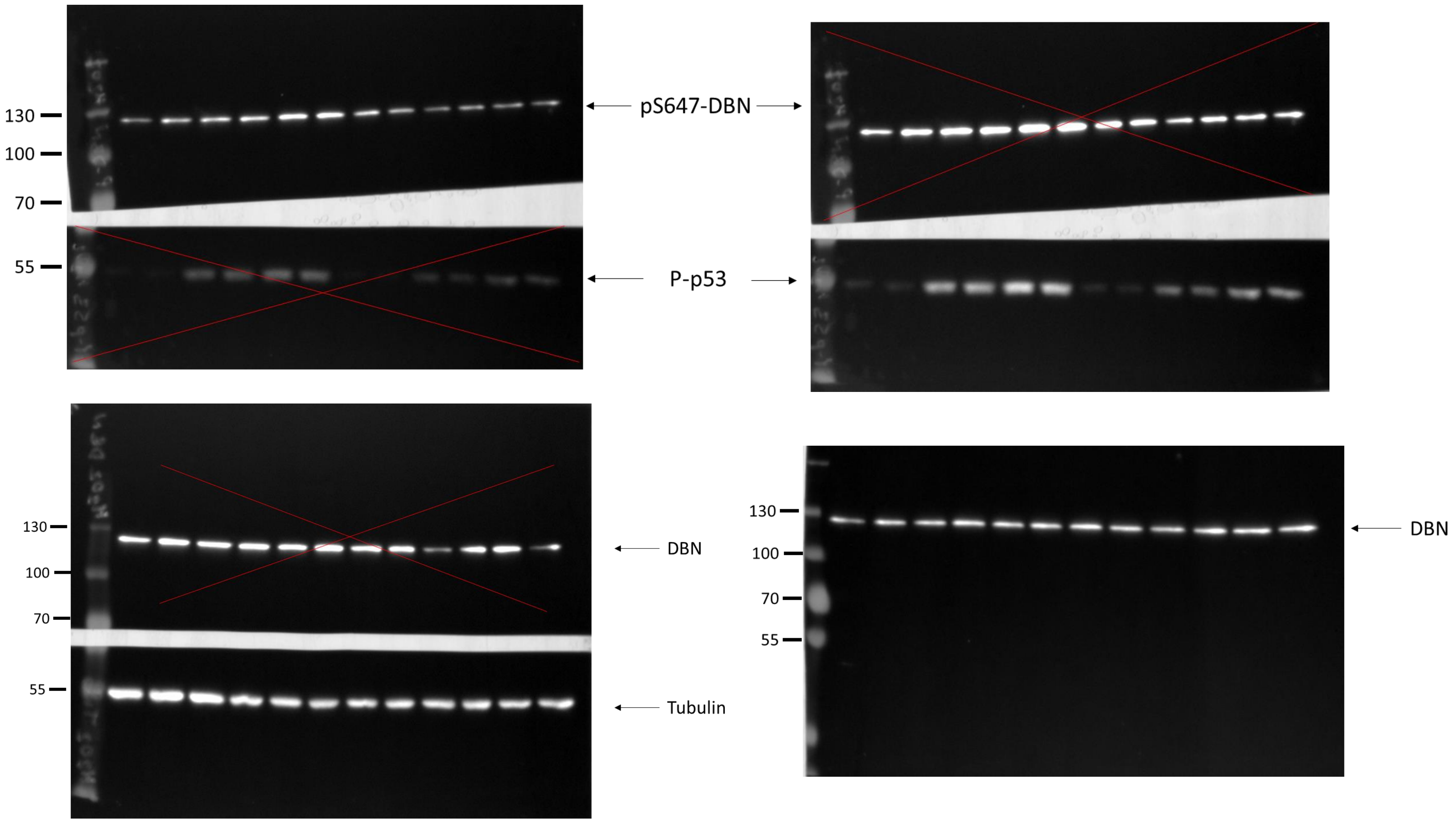

Supplementary figure 7. Uncropped western blots related to Figure 4c

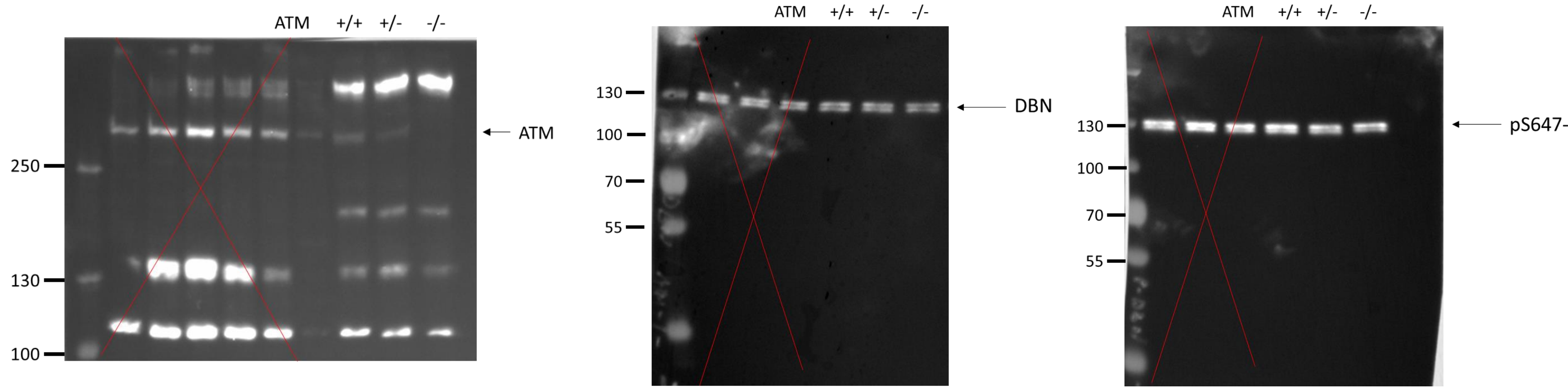

Supplementary figure 8. Uncropped western blots related to Figure 5d

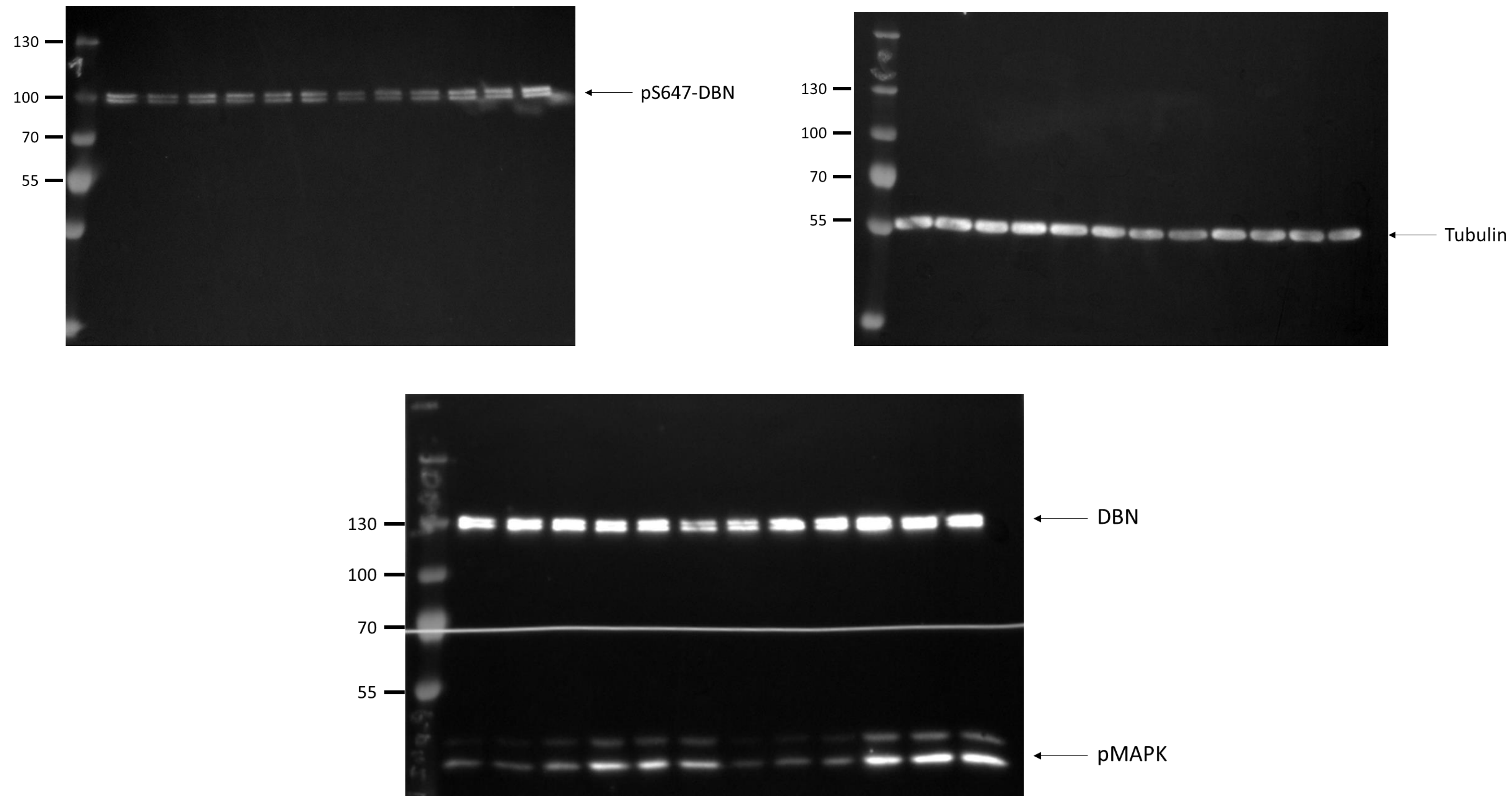

Supplementary figure 9. Uncropped western blots related to Figure 5e
